# Association between blood eosinophil count and Duchenne muscular dystrophy severity and prognosis: a retrospective cohort study

**DOI:** 10.1186/s13052-023-01483-y

**Published:** 2023-07-14

**Authors:** Zhi Jiang, Hongmei Liao, Liwen Wu, Wenjing Hu, Liming Yang, Bo Chen, Zeshu Ning, Jingwen Tang, Rong Xu, Mei Chen, Feng Guo, Shulei Liu

**Affiliations:** Departmentof Neurology, Hunan Children’s Hospital, Yuhua District, No.86, Zi Yuan Road, Changsha, 410007 China

**Keywords:** Eosinophils, Duchenne muscular dystrophy, Inflammation,Prognosis, Therapeutic efficacy

## Abstract

**Background:**

Duchenne muscular dystrophy (DMD) is a rare hereditary muscular disease. The role of eosinophils in DMD has not been clarified. This study aims to evaluate the association between peripheral blood eosinophil count and severity and prognosis of DMD.

**Methods:**

A retrospective cohort study was performed for 145 DMD patients between January 2012 and December 2020. Clinical data of 150 healthy children were collected as a control group. Logistic regression and Cox regression analyses were used to explore the influences of eosinophil count on DMD severity and prognosis.

**Results:**

Eosinophil count in DMD group was lower than the control group (Z = 2.163*, P =* 0.031). It was negatively correlated with Vignos scale score, Spearman correlation coefficient was *p =* 0.245*, P =* 0.040 (at admission)*,* *p =* 0.137*, P =* 0.032 (at follow-up); was a protective factor for high Vignos scale score at admission [odds ratio (OR) = 0.038, 95%CI: 0.002–0.752*, P =* 0.032] and follow-up (OR = 0.033,95%CI: 0.001–0.121*, P =* 0.039). The Cox regression analysis indicated that elevated eosinophil count was correlated with better therapeutic efficacy for DMD patients [hazard ratio (HR) = 2.218*,* 95%CI: 1.154–3.924, *P =* 0.016].

**Conclusion:**

Eosinophil count in peripheral blood was correlated with the severity of DMD. It could indicate the therapeutic efficacy and prognosis of DMD patients to a certain extent. Eosinophils may be a potentially valuable biomarker or therapeutic target for DMD.

## Background

Duchenne muscular dystrophy (DMD) is a severe X-linked disease characterized by progressive muscle weakness [1, 2]. Treatment of DMD is always a clinical challenge. The pathology of DMD involves gene abnormality, oxidative stress [3–5]. Inflammation, metabolic abnormalities, autophagy and regeneration defects are the major causes of it [6]. Inflammation plays a crucial role in the progression of DMD. Regulating inflammatory response and inducing immune tolerance to expression of dystrophin are key to treatment of DMD [7]. Nuclear factor-κB (NF-κB) signaling pathway promotes inflammation and limits muscle regeneration in DMD, resulting in fibrosis and fatty tissue replacement of muscle [8]. Increased levels of proinflammatory factors in DMD patients have been demonstrated. Inhibition of the inflammatory response contributes to improving muscle strength and outcome of DMD patients [3, 9].

Eosinophils contribute to initiation and modulation inflammation.

It may be caused by allergy, infectious, inflammatory, neoplastic disorders. Eosinophils regulate immune homeostasis, inhibiting proinflammatory response of overreaction by secreting specific molecules [10]. Eosinophils in peripheral blood are associated with the prognosis of chronic obstructive pulmonary disease (COPD), infectious diseases, several cancer [11–13].

Study confirmed eosinophil infiltration in muscle tissue of DMD patients. It can promote repair muscle cells [14]. On the other hand, persistence of eosinophils within dystrophic muscle would sustain fibroblast proliferation, which promotes fibrosis tissue deposition, accelerating clinical decline of disease [2]. The role of eosinophils in DMD is still controversial and unclear. Few studies to explore whether it affects long-term outcome of DMD, to date. The present study aimed to assess the association between the eosinophil count in peripheral blood and the severity and prognosis of DMD.

## Methods

### Study design

Our cohort comprised children with DMD from Hunan Children’s Hospital (Changsha, China) from January 1, 2012 to December 31, 2020. The inclusion criteria were as follows. 1) Boys aged within 2–5 years old (or slightly lower than 2 years old) with typical clinic-al symptoms. 2) Confirmation of DMD by genetic testing. 3) They had not been dealt at admission. The exclusion criteria were as follows: 1) Genetic testing exclude DMD. 2) Basic diseases, including infection, diarrhea, allergy, immune deficiency disease, or blood system disease. 3) Children with incomplete follow-up data. 4) Follow up was not completed. In total, 150 healthy children were enrolled as a control group. The inclusion criteria for the control group were as follows: 1) Healthy boys without obvious diseases during 3 months of follow-up. 2) Voluntary participation in vaccination or physical examination in our hospital. 3) Healthy volunteers participating in the value-driven health plan for testing of blood and other biochemical indicators in our hospital and Beijing Children’s Hospital. The exclusion criteria for the control group were as follows: 1) Combined with some diseases, such as infection, diarrhea, allergy. 2) Children with developmental or metabolic abnormalities. 3) Children with diseases that might influence the clinical data. 4) Study withdrawal due to various reasons. The present study was approved by the Ethics Committee of Hunan Children’s Hospital (Approval No. KS2022-15).

### Data collection

Clinical data were collected for age and muscle strength score (Vignos scale [3]). All laboratory data, such as blood routine and myocardial enzyme spectrum were collected. While vitamin D3 (at admission, *n =* 55; at follow-up, *n =* 46, note: vitamin D examination of the control group and case group was matched by season) and C-reactive protein (CRP; at admission, *n =* 62; at follow-up, *n =* 41) were collected only from some children. All blood routine and biochemical tests were performed at 6:30–8:30 a.m.

### Treatment

1. Conservative treatment (*n =* 50): When children’s parents knowing DMD was incurable, some parents declined prednisone treatment and selected conservative treatment, such as fructose sodium diphosphate and coenzyme Q10 supplement. A few children’s parents did not decide whether receive conservative treatment during the treatment period, while they were followed up regularly (*n =* 6).
2. Prednisone therapy (*n =* 95): Children aged within 5 years old received prednisone monotherapy (0.75 mg/kg/d QD), some children slightly late received this drug. Children were routinely supplemented calcium and vitamin D.

### Follow-up

Patients were followed up regularly after hospital discharge. They returned to the outpatient clinic of our hospital every 3–6 months to evaluate muscle strength and disease progression. The follow-up was terminated on April 30, 2021. The adverse reactions of prednisone and other drugs were monitored regularly. The therapeutic effects were evaluated at the last follow-up, the blood routine, myocardial enzymes were rechecked.

### Statistical analysis

Normally distributed data were expressed as mean ± standard deviation. Data with skewed distribution were presented as median (P25,P75). Normally distributed data were analyzed by independent-samples t-test or paired t-test. The Mann–Whitney U test was utilized for analysis of abnormally distributed data. Pearson correlation analysis was used for assessment of the relationship between normally distributed data, Spearman correlation analysis was employed for abnormally distributed data. The logistic regression analysis was applied to evaluate the risk factors. The Kaplan–Meier analysis and Cox regression analysis were utilized to explore effects of various factors on the treatment efficacy. All data were processed using SPSS 24.0 software (IBM, Armonk, NY, USA). *P* < 0.05 was considered statistically significant.

## Results

### Patient clinical profiles

**Fig. 1:**
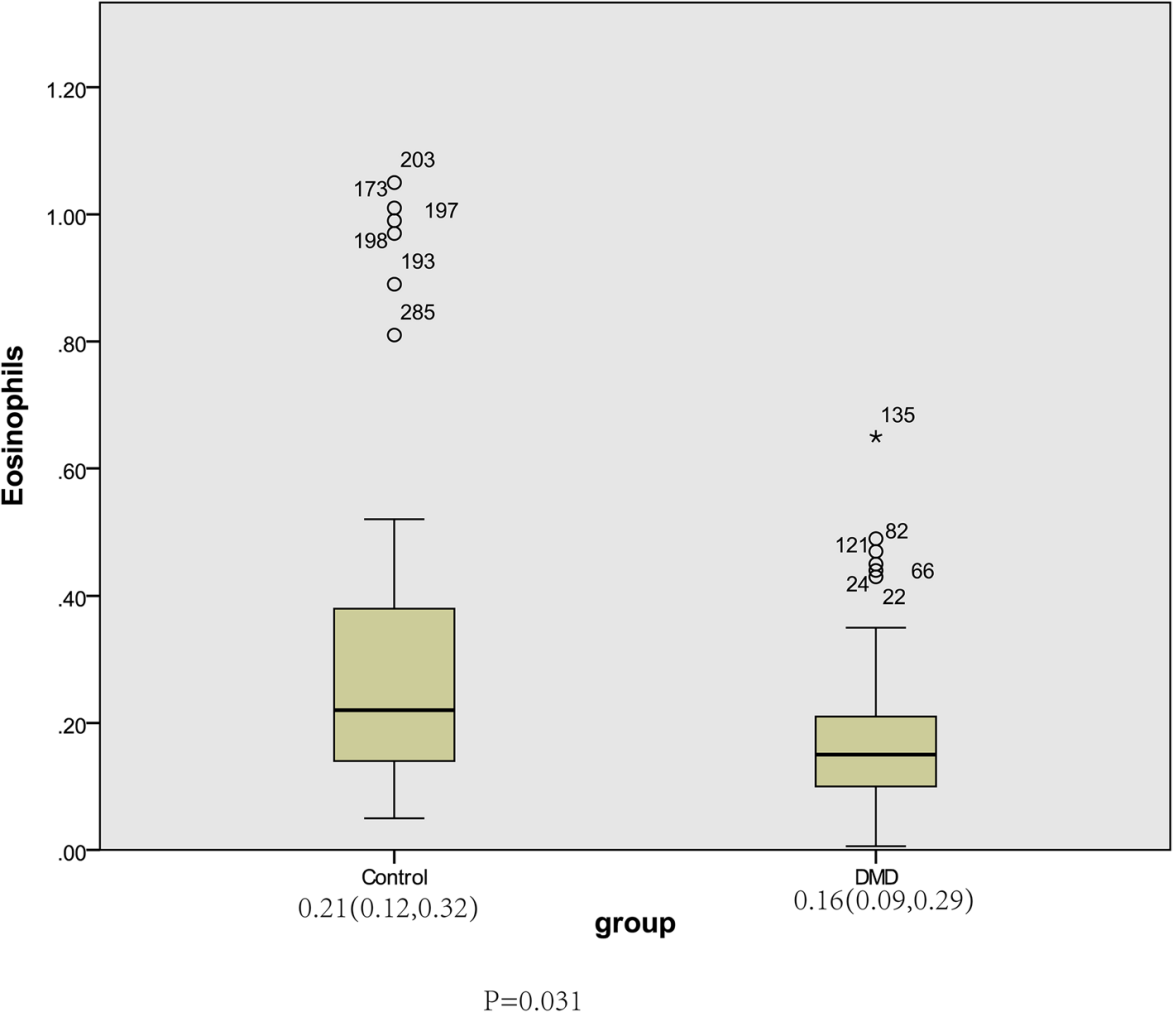
Different eosinophil count between DMD and control group

Among 287 children were primarily enrolled, 80 cases did not complete blood routine and muscle enzyme tests. Forty cases lost follow-up because of not followed up on time or changed contact information. Twenty-two cases withdrew from the study because of pulmonary infection or diarrhea during treatment or follow-up. Thus, 145 children were involved in our cohort, and 150 children were included in the control group. The eosinophil level was 0 for eosinophil count of 0–0.16 × 10^9^/l. It was 1 when eosinophil count ≥ 0.16 × 10^9^/l. The results of genetic testing showed that 145 cases completed genetic testing, including 92 cases of gene deletion (80 cases of large fragments and 12 cases of small fragments), 22 cases of gene duplication and variation (4 cases of single-exon duplication and 16 cases of multi-exon duplication), 31 cases of point mutation (4missense variants, 16 nonsense variants, 6 frameshift variants, 5 splice variants). The mean follow-up time in the DMD group was 1.67 (0.50, 3.25) years. The detailed information of DMD and control group is shown in Table 1. The number of eosinophils in the DMD group was significantly lower than that in control group (Fig. 1).

**Table 1:** General characteristics of the DMD and control group

	DMD group *N =* 145	Control group *N =* 150	Value	*P*
Age (years)	3.83 (1.42,6.08)	2.92 (1.17,5.92)	-0.795	0.427
Eosinophil count(× 10^9^/l)	0.16 (0.09, 0.29)	0.21 (0.12, 0.32)	2.163	0.031
Ratio of eosinophils	0.02 (0.01,0.03)	0.03 (0.02,0.05)	3.454	0.001
White blood cell count(× 10^9^/l)	8.00 (6.39, 9.82)	7.55 (6.40, 9.41)	0.880	0.379
Neutrophil count(× 10^9^/l)	3.43 (2.52, 4.36)	2.87 (2.30, 3.86)	2.723	0.006
Lymphocyte count(× 10^9^/l)	3.34 (2.69, 4.03)	3.47 (2.87, 4.73)	-1.407	0.159
Monocyte count(× 10^9^/l)	0.35 (0.27, 0.49)	0.41 (0.32, 0.59)	-2.896	0.004
Red blood cell count(× 10^12^/l)	4.65 ± 0.37	4.79 ± 0.39	3.060	0.002
Hemoglobin(g/l)	122.83 ± 10.29	128.51 ± 9.89	4.651	0.000
Basophil count(× 10^9^/l)	0.01 (0.003, 0.02)	0.01 (0.002, 0.03)	0.940	0.347
Platelet count(× 10^9^/l)	311.24 ± 92.23	308.32 ± 65.48	0.297	0.767
Vitamin D3(nmol/l)	47.91 ± 11.48	82.60 ± 23.12	6.439	0.000
Total protein(g/l)	66.18 ± 4.13	68.66 ± 4.41	4.820	0.000
Albumin(g/l)	42.52 ± 3.71	46.74 ± 2.39	11.042	0.000
Globulin(g/l)	23.67 ± 3.87	21.93 ± 3.02	4.193	0.000
Creatinine (umol/l)	18.41 ± 7.79	35.25 ± 10.18	13.375	0.000
NLR	1.04 (0.71,1.55)	0.87 (0.60,1.21)	2.535	0.011

*NLR* neutropil-to-lymphocyte ratio

One hundred forty-five children with DMD completed follow-up, in all. Comparedwith data collected at admission, the eosinophil count, erythrocyte level, hemoglobin level at follow-up were significantly different (*P* < 0.05 or 0.01). The results are shown in Table 2.

**Table 2:** Clinical characteristics of DMD patients at admission and follow-up

	cases at admission (*N =* 145)	cases at follow-up (*N =* 145)	Value	*P*
Age (years)	3.83 (1.42,6.08)	5.25 (3.16,7.16)	10.449	0.000
Eosinophil count(× 10^9^/l)	0.16 (0.09,0.29)	0.11 (0.07,0.19)	2.251	0.032
Ratio of eosinophils	0.02 (0.01,0.03)	0.02 (0.01,0.03)	1.105	0.269
White blood cell count(× 10^9^/l)	8.00 (6.39,9.82)	7.58 (6.16,8.87)	0.421?	0.674?
Basophil count(× 10^9^/l)	0.01 (0.003,0.02)	0.01 (0.01,0.02)	0.766	0.444
Redblood cell count(× 10^12^/l)	4.65 ± 0.37	4.85 ± 0.42	4.577	0.000
Hemoglobin(g/l)	122.83 ± 10.29	129.99 ± 12.50	5.521	0.000
Neutrophil count(× 10^9^/l)	3.43 (2.52,4.36)	3.19 (2.71,4.41)	0.895	0.371
Lymphocyte count(× 10^9^/l)	3.34 (2.69,4.03)	3.51 (2.51,4.27)	0.330	0.741
Monocyte count(× 10^9^/l)	0.35 (0.27,0.49)	0.40 (0.33,0.52)	-1.108	0.268
Platelet count(× 10^9^/l)	311.24 ± 92.23	299.76 ± 66.14	1.079	0.284
Vitamin D3(nmol/l)	47.91 ± 11.48	45.22 ± 9.23	1.155	0.307
Vignos scale	2 (1,3)	2 (1,3)	0.780	0.435
CK(U/L)	4177.00 (1698.70,9831.30)	7108.30 (1993.20,11,755.30)	2.074	0.038
Total protein(g/l)	66.18 ± 4.13	66.24 ± 4.29	0.425	0.672
Albumin(g/l)	42.52 ± 3.71	42.62 ± 3.35	0.049	0.961
Globulin(g/l)	23.67 ± 3.87	23.62 ± 2.97	0.398	0.692
CRP (mg/l)	0.75 (0.18,1.21)	0.50 (0.30,0.84)	1.521	0.128
Creatinine (umol/l)	18.41 ± 7.79	23.41 ± 9.17	2.933	0.006
NLR	1.02 (0.71,1.55)	0.95 (0.76,1.47)	1.333	0.182

*CK* Creatinekinase; *CRP* C-reactive protein; *NLR* neutropil-to-lymphocyte ratio

### Correlation analysis

There were correlations between eosinophil count and vitamin D3level, neutropil-to-lymphocyte ratio (NLR) in the control group (Spearman correlation coefficient *p =* 0.299, *P =* 0.012, *p =* -0.261, *P =* 0.018). The correlation coefficient between eosinophil count and Vitamin D3 level in DMD group at admission was *p =* 0.563 (*P =* 0.001), at follow-up was *p =* 0.267(*P =* 0.207).

There were correlations between the eosinophil count and CRP level, NLR at admission (*p =* 0.271, *P =* 0.046, *p =* 0.232, *P =* 0.027), at follow-up (*p =* 0.189, *P =* 0.035, *p =* 0.227, *P =* 0.023) in DMD group.

Correlation coefficient between eosinophil count and Vignos scale score at admission was *p =* 0.245 (*P =* 0.040). Patients were divided into conservative treatment group (*n =* 50) and prednisone treatment group (*n =* 95). In prednisone treatment group, the correlation coefficient between eosinophil count and Vignos scale score at admission was *p =* 0.259 (*P =* 0.041), at follow-up was *p =* 0.092 (*P =* 0.023). In conservative treatment group, correlation coefficient between eosinophil count and Vignos scale score at admission was *p =* 0.212 (*P =* 0.043)*,* at follow-up was *p =* 0.233 (*P =* 0.004)*.*

Correlation coefficient between lymphocyte count and Vignos scale score at admission was *p =* 0.257, (*P =* 0.004), at follow-up was *p =* 0.195(*P =* 0.032). The correlation coefficient between eosinophil count and lymphocyte count, monocyte count at admission was *p =* 0.244 (*P =* 0.007), *p =* 0.182 (*P =* 0.044); at follow-up was *p =* 0.038 (*P =* 0.7160), *p =* 0.46 (*P =* 0.656).

### Effects of treatment

In prednisone treatment group, eosinophil count was 0.15 (0.08, 0.25) × 10^9^/l at time of treatment and 0.08 (0.05,0.17) × 10^9^/l at follow-up. The eosinophil count was different statistically before and after treatment (Z = 3.157, *P =* 0.002).

The eosinophil count in the conservative treatment group was 0.19 (0.10, 0.31) × 10^9^/l at admission, and 0.20 (0.11, 0.27) × 10^9^/l at follow-up.

The eosinophil count was no difference before and after treatment (Z = 0.270, *P =* 0.787), statistically. The eosinophil count in conservative treatment group and prednisone treatment group was compared with that before treatment (Z = 1.464, *P =* 0.143), in which there was no statistically significant difference, while there was a significant difference after treatment (Z = 4.559,*P =* 0.000).

### Eosinophil affected muscle strength

Single-factor regression analysis of Vignos scale score in patients with DMD at admission revealed that age at admission, creatinine level, lymphocyte count, mononuclear count, eosinophil count was statistically significant. Age was a risk factor, while others were protective factors for muscle strength of DMD patients (Table 3).

**Table 3:** Influencing factors for muscle strength score in DMD patients at admission

	Univariate analysis			Multivariate analysis		
	OR	95%CI	*P*	OR	95%CI	*P*
Age	1.453	1.180–1.789	0.000	2.619	1.369–5.003	0.004
Lymphocyte count	0.646	0.434–0.960	0.031	0.531	0.237–1.190	0.124
Eosinophil count	0.007	0.001–0.276	0.008	0.038	0.002–0.752	0.032
Creatinine	0.865	0.754–0.953	0.039	0.950	0.889–1.106	0.135
Monocyte count	0.046	0.003–0.849	0.038	0.191	0.022–1.653	0.133

*OR* odds ratio; *CI* Confidence interval

In multivariate logistic regression analysis, we found that the eosinophil count [odds ratio (OR) = 0.038, 95% confidence interval (CI): 0.002–0.752, *P =* 0.032] was a protective factor for muscle strength score. The higher eosinophil count, the lower the score, and the higher muscle strength. Results of logistic regression analysis of factors influencing muscle strength at admission are shown in Table 3.

The univariate analysis of Vignos scale score at follow-up revealed that age at follow-up, treatment, lymphocyte count, monocyte count, and Vignos scale score at admission were statistically significant. The eosinophil count at follow-up and at admission was also found statistically significant.

Further controlling of age and other factors performed by the multivariate regression analysis revealed the effects of treatment method (OR = 0.167, 95%CI: 0.030–0.931, *P =* 0.041) and Vignos scale score at admission (OR = 13.582, 95% CI: 1.332–138.492, *P =* 0.028). The effects of eosinophil count at follow-up on muscle strength score were statistically significant (OR = 0.033, 95%CI: 0.001–0.821, *P =* 0.039). Results of logistic regression analysis of factors influencing muscle strength during follow-up are presented in Table 4.

**Table 4:** Influencing factors for muscle strength score in DMD patients during follow-up

	Univariate analysis			Multivariate analysis		
	OR	95%CI	*P*	OR	95%CI	*P*
Age	1.334	1.134–1.570	0.000	2.834	0.831–9.668	0.096
Treatment	0.404	0.178–0.918	0.030	0.167	0.030–0.931	0.041
Lymphocyte count	0.630	0.439–0.904	0.012	0.767	0.568–1.035	0.083
Monocyte count	0.015	0.001–0.405	0.013	0.169	0.016–1.739	0.135
Vignos scale score at admission	11.543	5.607–26.297	0.000	13.582	1.332–138.492	0.028
Eosinophil count at follow-up	0.012	0.001–0.645	0.029	0.033	0.001–0.821	0.039
Eosinophil count at admission	0.016	0.002–0.586	0.024	0.002	0.001–20.452	0.167

*OR* odds ratio; *CI* Confidence interval

### Eosinophil affect therapeutic efficacy

The results of univariate Cox regression analysis revealed that age [hazard ratio (HR) = 0.851, 95%CI: 0.771–0.939, *P =* 0.001], treatment modality (HR = 3.362, 95%CI:1.222–5.607, *P =* 0.000)*,* Vignos scale score (HR = 0.637, 95%CI: 0.454–0.863, *P =* 0.002), lymphocyte count (HR = 1.056, 95%CI: 1.003–1.111, *P =* 0.037), granulocyte count (HR = 0.785, 95%CI: 0.457–0.954, *P =* 0.048), albumin level (HR = 1.131, 95%CI: 1.060–1.206, *P =* 0.002) at admission; granulocyte count (HR = 0.481, 95% CI: 0.254–0.912, *P =* 0.025) and eosinophil count at follow-up (HR = 1.895, 95%CI: 1.313–2.733, *P =* 0.001) were statistically significant.

The multivariate regression analysis revealed that age (HR = 0.929, 95% CI: 0.869–0.933, *P =* 0.031) and Vignos scale score (HR = 0.484, 95%CI: 0.267–0.880, *P =* 0.017) at admission, treatment modality (HR = 4.174, 95%CI: 2.401–7.495, *P =* 0.003), eosinophil count at follow-up (HR = 2.218, 95%CI: 1.154–3.924, *P =* 0.016) were statistically significant. The results suggested that the higher eosinophil count, the better therapeutic efficacy. These results are shown in Table 5.

**Table 5:** Influencing fators for therapeutic efficacy of DMD patients

	Univariate Cox regression			Multivariate Cox regression		
	HR	95%CI	*P*	HR	95%CI	*P*
Age atadmission	0.851	0.771–0.939	0.001	0.929	0.869–0.993	0.031
Treatment modality	3.362	1.222–5.607	0.000	4.174	2.401–7.495	0.003
Vignos scale score at admission	0.637	0.454–0.863	0.002	0.484	0.267–0.880	0.017
Lymphocyte count at admission	1.056	1.003–1.111	0.037	1.232	0.949–1.613	0.117
Granulocyte count at admission	0.785	0.457–0.954	0.048	0.604	0.278–1.295	0.174
Granulocyte count at follow-up	0.481	0.254–0.912	0.025	0.611	0.285–1.439	0.198
Albumin at admission	1.131	1.060–1.206	0.002	1.250	0.941–1.643	0.109
Eosinophil count at follow-up	1.895	1.313–2.733	0.001	2.218	1.154–3.924	0.016
Eosinophil count at admission	1.281	0.743–1.572	0.684	1.147	0.904–1.463	0.164

*HR* Hazard ratio; *CI* Confidence interval

**Fig. 2:**
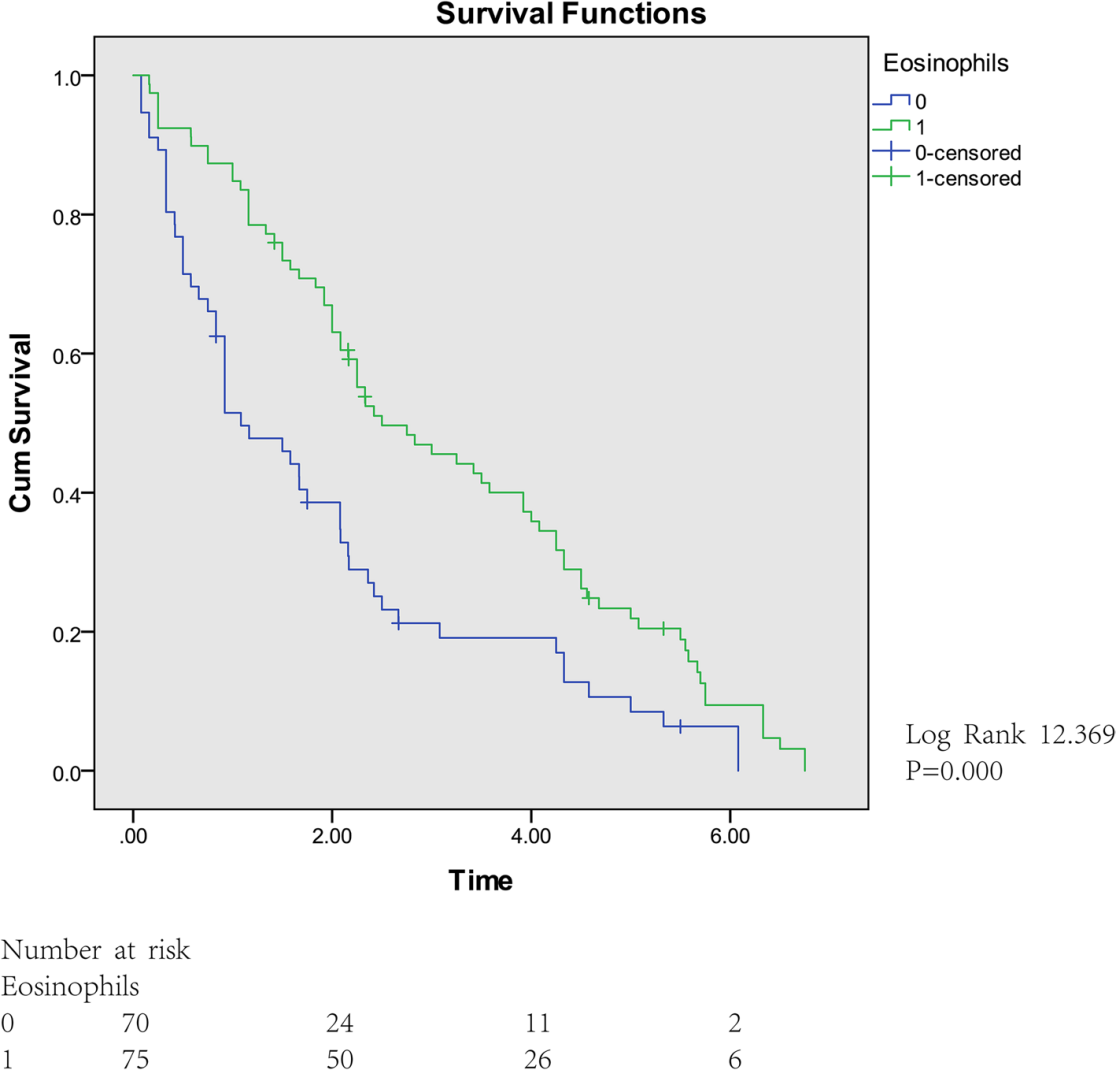
Survival curve for time to muscle strength decreased according to eosinophil count in DMD patients

Kaplan–Meier analysis of eosinophil count at follow-up showed that there were significant differences in different eosinophil count on survival time of children with DMD. The median survival time of 0 level was 1.083 years, standard deviation was 0.273, while that of 1 level was 2.524 years, standard deviation was 0.519 (Fig. 2).

**Fig. 3:**
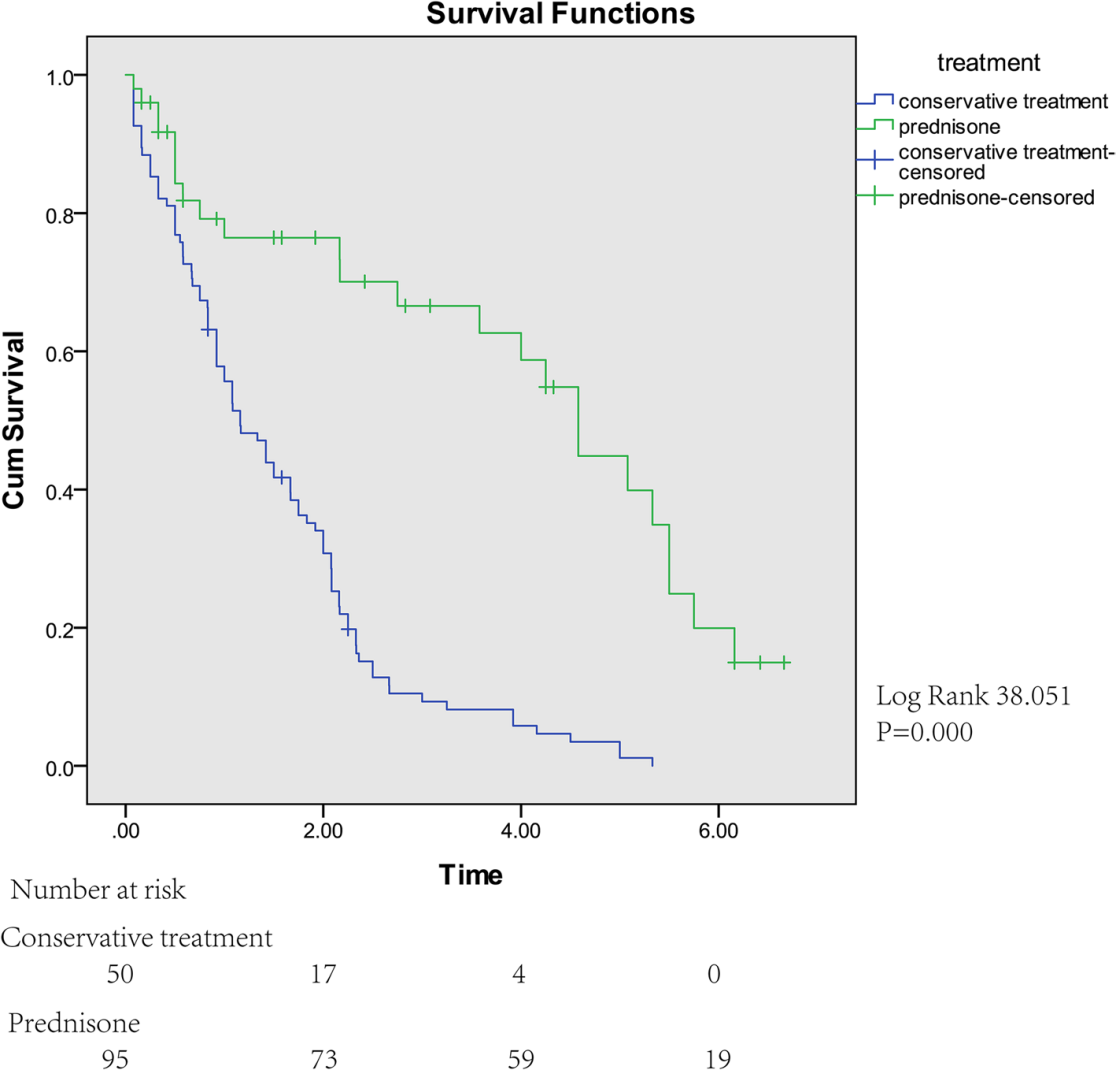
Survival curve for time to muscle strength decreased according to treatment modalities in DMD patients

The higher eosinophil count, the longer survival time of children with DMD. There were also significant differences in effects of different treatment modalities on survival time of children with DMD during follow-up. The median survival time in the conservative treatment group was 1.160 years, standard deviation was 0.184; the median survival time in prednisone treatment group was 4.580 years, standard deviation was 0.405 (Fig. 3). The therapeutic efficacy of prednisone treatment was significantly higher than conservative treatment. (To evaluate the therapeutic efficacy more conveniently, we considered that the treatment was ineffective if the Vignos scale score increased. If survival time was long, the therapeutic efficacy would be better).

**Fig. 4:**
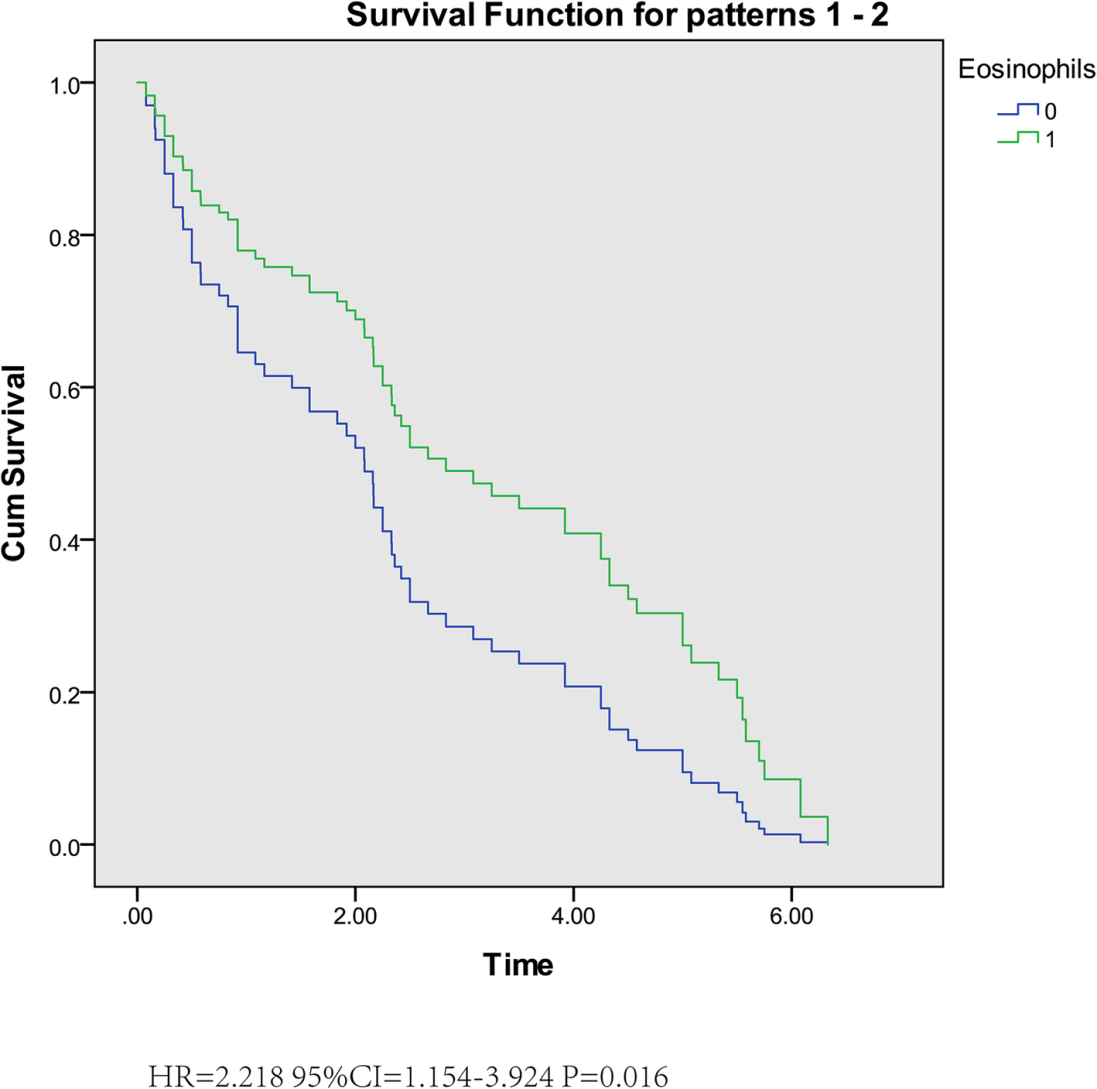
Cox regression for independent factors on muscle strength decreased in DMD children after adjustment. Abbreviations: HR: hazard ratio CI: confidence interval

In the Cox regression analysis, there were significant differences in the therapeutic efficacy of different eosinophil count on children with DMD (Fig. 4). The higher the eosinophil count, the better the therapeutic efficacy.

## Discussion

We demonstrated that the eosinophil count was related to muscle strength and long-term treatment effect in DMD patients. Eosinophilcount was negatively correlated with the intensity of inflammatory response in vivo. These may stem from the immunoregulatory and muscle repair of eosinophils.

Growing evidence proved that inflammation influences DMD. Continuous inflammatory response aggravates severity of DMD [3, 9]. Chronic inflammation is a pathological feature of DMD. DMD patients are accompanied with the elevated levels of inflammatory factors, such asinterleukin-6 (IL-6), interferon-γ (IFN-γ), tumor necrosis factor-α (TNF-α), etc. The effectiveness of prednisone treatment is also a strong evidence of an inflammatory response.

There is a chronic inflammatory response in children with DMD. Eosinophils are engaged in immune inflammatory response. We confirmed that there was a certain correlation between eosinophil count and muscle strength score. It confirmed that eosinophil count had a definite impact on the muscle strength. We found that eosinophil count in children with DMD was negatively correlated with CRP level and neutropil-to-lymphocyte ratio (NLR). NLR is a simple inflammatory marker and is related to the prognosis of many diseases such as infection, tumor and immunity [15]. Therefore, eosinophil count was negatively correlated with systemic inflammatory response. Eosinophil can downregulate the inflammatory response. In our study, eosinophil count in the control group was higher than DMD group. This showed that the control group has higher ability to downregulate inflammatory response.

It is widely accepted that eosinophils are inflammatory cell, which is related to allergy and parasitic infection. The increase of eosinophil count represents an inflammatory response. Why is it negatively correlated with CRP level ? A COPD study confirmed that the eosinophil count in peripheral blood was negatively correlated with CRP level. That could be related to the fact that eosinophils enter the lung tissue when the inflammatory reaction is obvious, leading to eosinophils decreased [16]. We speculate that there is a similar reason, in which eosinophils enter muscle tissue, thus, eosinophil count in peripheral blood in DMD group was slightly lower than the control group. Eosinophils can maintain Th1/Th2 (T helper1/T helper2) immune balance and inhibit excessive inflammatory response in vivo [17]. We believe that eosinophils inhibit the excessive inflammatory response in children with DMD. However, this needs more research to confirm it.

Our study showed that a high eosinophil count means better muscle strength and therapeutic efficacy (Fig. 2). However, previous study demonstrated that eosinophil count in peripheral blood was not related to eosinophil count in DMD muscle [18]. The relationship between its change and disease severity cannot be compared with the relationship between number of eosinophils in muscle and disease severity.

Our study revealed that the eosinophil count was negatively correlated with Vignos scale score before and after prednisone treatment, although it was very weak. This correlation persisted in the conservative treatment group. We found that eosinophils have a certain protective effect on muscle strength of DMD patients. However, the underlying mechanism should be further studied.

We concluded that eosinophils were protective factors for high motor function score whether at admission or at follow-up. After treatment, we found that the lower eosinophil count in prednisone treatment group, the worse therapeutic efficacy. Our Kaplan–Meier analysis and multivariate Cox regression analysis also supported this conclusion. The effects of eosinophil count on therapeutic efficacy of DMD might originate from its protective effects on muscle strength. Animal experiments demonstrated that eosinophils do not mediate the acute muscle injury [19], but has not been confirmed in human experiments. The present study showed that a high eosinophil count contributes to the improvement of long-term therapeutic efficacy of DMD. This may indirectly confirm that eosinophils play a certain role in repairing muscle damage in DMD patients [10]. However, this finding needs to be further verified.

Vitamin D regulates calcium and phosphorus metabolism as well as the inflammatory response. Its immunomodulatory ability has been used to treat multiple sclerosis and systemic lupus erythematous (SLE) [20, 21]. Vitamin D3 regulates the inflammatory response through the vitamin D receptor, enhance T regulatory (Treg) cell function, induce immune tolerance.

Our study confirmed that level of vitamin D3 in the DMD group was significantly lower than control group. Thus, the ability of DMD group to regulate the inflammatory response was relatively low. Our study revealed that the number of eosinophils was positively correlated with vitamin D3 level. Literature confirmed eosinophils inhibit excessive inflammatory response in vivo [17]. We speculate that eosinophils have similar ability to downregulate inflammatory responses as vitamin D3. The stronger correlation between eosinophil count and vitamin D3 level in DMD group was due to lower vitamin D3 level and lower eosinophil count in DMD group than control group.

Eosinophils are regulated by lymphocytes and their cytokines. T lymphocytes are involved in inflammatory response of DMD. Previous studies demonstrated that number of T cells decreased after prednisone treatment in DMD patients, which was accompanied by reduced muscle necrosis and fibrosis. Our study indicated that blood lymphocytes were protective factor of muscle strength scores at admission. However, multivariate Cox regression analysis showed no statistical significance. This could be due to the small sample size or other factors. Previous study reported that the number of CD8/CD26T cells in peripheral blood of DMD patients was positively correlated with quantitative muscle score (i.e., the higher number, the greater muscle strength), which is consistent with our results [22]. Another research demonstrated that CD49d can be used as a marker and potential therapeutic target for DMD [23].

Eosinophils are activated by monocytes, which participating inflammatory response of DMD. Monocyte macrophages appear in the early-stage of muscle injury, in which pro-inflammatory macrophages mainly mediate the inflammatory response, and pro-regenerative macrophages inhibit this response to repair muscle cells [24]. Animal experiments have shown that inflammatory monocytes improve the prognosis of patients with DMD, and may play a role through chemokine receptor-2 (CCR2) [25]. Our study confirmed that the eosinophil count was correlated with monocyte changes. This may suggest that the role of eosinophils is similar.

Influence of age on Vignos scale score is obvious. For DMD, the deletion of dystrophin protein is caused genetically. With the increase of age, muscle damage, degradation, children with DMD may lose motor function at about 12 years old if do not timely receive treatment. With increase in age, the Vignos scale score gradually increases, which are consistent with our results. In our study, Cox regression analysis revealed that therapeutic efficacy was reduced with the increase of age. With increase in age, the interaction between chronic activation of innate immunity and degradation and regeneration of asynchrony and proximity produced an uncoordinated repair response. This repair response could promote the disease progression.

At present, effective treatment for DMD is prednisone, while its adverse reactions are noteworthy. To our knowledge, difecolate is more effective than prednisone (we did not use this drug in our study because few patients preferred to receive it) [26]. Although the treatment mechanism of prednisone for DMD has not been fully clarified [27, 28], our study confirmed that prednisone could control the inflammatory response. Our logistic regression suggested that prednisone had a protective effect on muscle strength. Muscle strength could be improved with prednisone treatment. Our Cox regression analysis further confirmed that prednisone was effective, which was consistent with previously reported findings [29].

We found eosinophil count can not only reflect inflammatory response of DMD patients, but also represent muscle strength or prognosis. Because some children cannot complete muscle strength test or 6-min walking test well. It is important to seek a simple and practical biomarker to indicate the muscle strength or degree of muscle injury in DMD patients. Eosinophil count can represent muscle strength to some extent. We confirmed eosinophil count affects therapeutic efficacy of children with DMD, so it can be used as a therapeutic target.

There were some deficiencies in the present study. First, the sample size was small, and the 6-min walk test was not completed (some children did not cooperate). Second, follow-up time of DMD children was not long enough. Third, the association of eosinophil count and levels of cytokines in children with DMD was not assessed. Fourth, this study did not investigate specific mechanisms. We need animal experiments to prove it.

## Conclusions

In conclusion, eosinophil count in peripheral blood of children with DMD could reflect muscle strength and inflammatory response, could be associated with therapeutic efficacy, could represent prognosis to a certain extent. Eosinophils may be a potentially valuable biomarker or therapeutic target for DMD.

## Data Availability

The data that support the findings of this study are available from the corresponding author upon reasonable request.

## Abbreviations

ORCID: Open Researcher and Contributor Identity Document
DMD: Duchenne muscular dystrophy
OR: Odds ratio
CI: Confidence interval
NF-κB: Nuclear factor-κB
COPD: Chronic obstructive pulmonary disease
CRP: C-reactive protein
QD: Quaque die
CK: Creatine kinase
NLR: Neutropil-to-lymphocyte ratio
IL-6: Interleukin-6
IFN-γ: Interferon-γ
TNF-α: Tumor necrosis factor-α
Th1: T helper1
Th2: T helper 2
SLE: Systemic lupus erythematous
Treg: T regulatory
CD8: Cluster of differentiation 8
CD26: Cluster of differentiation 26
QMT: Quantitative muscle score
CD49d: Cluster of differentiation 49d
CCR2: Chemokine receptor-2
